# Disintegration at the Cervical Border

**Published:** 1888-12

**Authors:** L. P. Bethel

**Affiliations:** Toledo, O.


### Disintegration at the Cervical Border.

BY L. P. BETHEL, D D S , TOLEDO, O.

[Read before the Ohio State Dental Society, Cincinnati, October, 16, 1888.]

It is a fact well known to most, if not all of the profession, that failures of proximal fillings, are, in a majority of cases, due to disintegration at the cervical border. Much has already been said and written upon this subject and many theories advanced. Theories founded upon good judgment, and of such a character as to satisfactorily explain, at times, the predisposing causes of disintegration at the cervical border of proximal fillings. Yet, no one theory is applicable in all cases, for the circumstances are so various. There are failures, at times, when every effort has been put forth to make a perfect filling, and all care maintained to avoid that imperfect manipulation or practice that in any way is liable to become a barrier to the preservation of the tooth.

Among the more important theories advanced are those of imperfect preparation of cavities ; imperfect manipulation of the filling material, at the cervical wall ; the injudicious use of the mallet, particularly the hand mallet ; the imperfect finishing of fillings at the cervical borders, and others. It can not truthfully be denied that, if a frail wall is left at the cervical border, or in fact any defective manipulation as above stated, becomes a predisposing cause to the destructive influence of decay producing agents, yet, with all due care in these and other things we are often unable to satisfactorily explain the cause of disintegration.

From all accounts, more trouble of this kind has been experienced since amalgams and cohesive golds have been so generally adopted as filling materials, and this has lead to the inquiry if attending circumstances may not be the cause of a physiological action, that, *at times,* becomes the predisposing cause of decay at the cervical border, namely, *absorption.*

These failures occur mostly where the decay has burrowed high on the tooth, and especially where the gum has receded. The cervical border in these cases is formed where the enamel is often thin and defective, or extends quite into the cement substance overlapping the enamel.

Dr. Black* has stated that "much of the cementum of man, especially about the necks of the teeth, when so stained as to show them clearly, seems almost as if made up of fibers." And also that "these are the principal fibers of the peridental membrane included in the cementum in its growth, and furnish the means of making a firm hold of the peridental membrane upon the root of the tooth. They are white, connective tissue fibers, the ends of which are included in the matrix of the cementum and in the most part calcified with it. They are in all respects like the residual fibers of bone and serve a similar purpose."

Cementum is analagous to bone in composition and density. Dentine differs from cement, practically, in that it contains about 5 per cent. more phosphate of lime which renders it harder as well as reduces the proportion of organic matter. Enamel is much harder and denser than either, and thus less easily penetrated. Having briefly considered the character of the substances forming the cervical wall in this location, let us turn our attention now to the filling materials.

It seems to be difficult to get an amalgam that will not change its shape after insertion in the cavity. Of course manipulation has more or less to do with this change, but we will not consider that point here. As inserted, amalgams seem generally to either contract or expand, more or less, upon hardening. As a result of contraction there is a pulling away of the material from the walls of the cavity, and although the breach be small, the decay producing agents may find a lodging place here, and their destructive influence is manifested. On the other hand, expansion of the amalgam must cause lateral pressure against the walls of the cavity, and this continued pressure may become an irritant. Cohesive gold, when heated, becomes harsh and unyielding. When malleted against the walls of the cavity, especially in hurried manipulation, it is liable to form an uneaven surface and when solidly wedged in this position may become a slight irritant, by means of pressure.

Nature in both cases will then be called upon to take some course of action. First, we may reason that she will relieve the

*Dental Review.

pressure and this perhaps by absorption. Dr. Black* states that "the clinical history of cases seems to show that a moderate degree of irritation or inflammatory action may hasten, or even be the condition of the beginning of many of the absorptions." Dr. Coleman says:† "Pressure if continued upon the soft tissues, will produce absorption, and even the bony structure will after a time yield to the same cause."

In case absorption does ensue, where will it first manifest itself? Naturally at the most vulnerable point, in this case the cervical border, as it contains more organic matter, and is less dense than the enamel. If the absorptive process begins it may, and often does, proceed quite beyond, just enough to relieve the pressure, and in this case may cause a breach.

It is true that nature reacts in cases of absorption and fills the breach with cementum unless some intervening substance interferes. If the absorptive process has been carried on to such an extent that the oral fluids are admitted into the breach, then nature is called upon to protect herself from these and makes the deposit short of the breach and thus we get a condition that greatly favors disintegration and caries.

On the other hand we notice few failures at the cervical border where non-cohesive gold, tin, or tin-and-gold are judiciously used as a filling material. If the absorptive theory, as here presented, proves true, then we may conclude that fewer failures occur from these materials because they are soft and yielding, conforming readily to the walls of the cavity and on this account do not cause pressure after insertion.

Unfortunately we have not had the materials at hand to make original investigation in this direction and what we have said in this paper is written upon a supposition, with the hope that it may be the means of bringing forth truth in the discussion.

DISCUSSION OF DR. BETHEL'S PAPER.

*Dr. Smith*—This is a subject of much importance to us, and it seems to me that the paper ought to receive some discussion. The failure of this operation at the cervical border depends very

*Dental Review.

†Coleman's Pathology.

largely on the manipulation, it depends greatly on the failure in manipulation in making a close joint in the filling, and a great many times from the lack of finishing out the border. Dr. Bethel, if I understand him correctly, puts the failure to a depression of the filling, and I understood him to say that you might have continuous pressure. You have no doubt pressure at that point in the manipulation of solids—gold or silver—and you might endanger the tissue and then follows the dissolving action of digestion. That idea has been set forth by Prof. Black, in referring to what he called erosion of the teeth. Why are teeth eroded, the surfaces removed, without the presence of the contamination from foreign matter, where teeth are thoroughly rubbed, yet we have this eroded surface, that has been spoken of by Dr. Black, arising from the marginal gum, from the stomach, and in that way dissolving the dentine. Whether he means after the filling has been introduced we have a continuous pressure, or whether the pressure is noticed at the time thereby impairing the tissue, and making it more readily acted on by the solvent.

*Dr. Taft.*—This is an important practical question, many operators tell us that they have very frequently failed at this point and I presume nearly all, perhaps all operators have as many failures at this point as anywhere else. The question has frequently been asked why is this? Some give one reason, others another. There are certain things, however, that will be plain to every one as bearing upon the question. In the first place the cervical borders in proximate fillings are so situated that foreign substances are liable to lodge and be retained till decomposition takes place. It is difficult to keep the proximal surfaces of the teeth free from offensive accumulations. It is frequently a very difficult matter to have the cervical borders well protected, and especially if the cavity extends well toward the root of the tooth. And then the formation of a cervical wall is attended with difficulty. It is a difficult point to properly form definitely and well. In many instances it is necessary to work by reflected light in operating upon these borders. The cervical borders of proximate cavities are more or less difficult of access in the different steps of the operation of filling; this accounts for some defective work and failures.

A thorough examination of proximate fillings, as they are usually presented, will reveal quite a variety of defects. In some cases it will be found that the form of the cervical wall or border is defective; either the decay not all removed, the edge left irregular and rough, or sharp at the edge, so that in the introduction of the filling it has been fractured. In many cases there is a failure in the adaptation and consolidation of the filling, and so the cavity is not protected. And again it will be found that the cavity has not been sufficiently filled to protect the tooth; and further it is often found that the cavity is over-filled, and the material permitted to over-lap on the tooth outside of the cavity, thus affording a ready lodgment for destructive agents, by which decay is soon set up. The indications are to avoid these defects by careful and skillful manipulation in the operation of filling.

The filling and tooth should be so dressed that a free and smooth space is made between the necks of the teeth, that they may be easily kept clean. Sometimes the enamel and cementum fails to cover the dentine at the neck of the tooth; such points are very subject to decay; filling a cavity of decay in the vicinity of such an exposed point, though perfectly done, will in nowise prevent the attack of decay, this may be in contact with the filling, or it may be at a distance from it. A diseased gum-margin is often the occasion of decay, especially on the proximate surfaces of the teeth, but the cervical portion of the lingual and buccal teeth are affected in the same way. Now if these various points receive the attention that their importance demands and the treatment they should have, there will be less occasion for complaint about failures in proximate fillings.

*Dr. Clancey.*—If the tooth-substance is cut away under the gum, in my experience the filling will last very much longer, and this trouble is not likely to occur. The difficulty of the operation, the time it takes to make it, the obscure position of the cervical border, and the side walls of the cavity, leaving an edge towards its neck has much to do with the failure of this work. The filling is not perfectly done, if it is perfectly done it is as perfectly finished at the cervical border as the upper portions of the tooth, which are easy to get at. I think we will have fewer failures if we cut away so that the lower part of the tooth at the cervical borders do not come close together, and if the gold is placed in properly, and the margins of the side walls of the cavity are covered, and if too much force is not used, the tissue will not be broken down, and I do not see why they should not last as long as in any other place. If it is necessary, take more time. I do not see the necessity of so many failures, as we hear about, and as I have had myself and have seen coming from the hands of other operators.

